# Soil-Transmitted Helminth Infections and Associated Risk Factors Among Primary Schoolchildren in the Koh Yao Islands of Southern Thailand

**DOI:** 10.1155/jotm/2907585

**Published:** 2025-07-24

**Authors:** Nonthapan Phasuk, Prasit Na-Ek, Udomsak Narkkul, Stephen J. Scholand, Chuchard Punsawad

**Affiliations:** ^1^School of Medicine, Walailak University, Nakhon Si Thammarat 80160, Thailand; ^2^Center of Excellence in Tropical Pathobiology, Walailak University, Nakhon Si Thammarat 80160, Thailand; ^3^Department of Medicine, University of Arizona, Tucson 85724, Arizona, USA

**Keywords:** hookworm infection, intestinal parasites, prevalence, schoolchildren, soil-transmitted helminths, Thailand *Trichuris trichiura*

## Abstract

Soil-transmitted helminth (STH) infections are the most common parasitic infections worldwide, particularly in tropical regions. There are currently no reports on the prevalence of STH infections among children in Koh Yao District of Phang Nga Province, Thailand. Therefore, this study aims to assess the prevalence of STH infections and associated risk factors among primary schoolchildren. A school-based cross-sectional study was conducted among 243 schoolchildren aged 7 to 12 years from 12 rural primary schools across three subdistricts. Demographic data and associated risk factors were collected and analyzed. Stool samples were collected and examined for parasites using the formalin-ethyl acetate sedimentation concentration and the modified Kato-Katz methods. A multivariate logistic regression analysis was used to determine the strength of the relationship between risk variables and STH infections. Of the 243 participants (mean age: 8.99 ± 1.57 years), 12 (4.94%, 95% CI: 2.60%–8.50%) were infected with STH. The most common STH infection was hookworm (4.11%), followed by *Trichuris trichiura* (1.65%). More than 80% (*n* = 10, 83.33%) had a single intestinal parasite, while two (16.67%) were coinfected with two parasitic species. Infections were more prevalent among males (66.67%) compared to females (33.33%). Multiple regression analyses revealed that children above Grade 3 had a significantly higher prevalence of STH infections compared to those below Grade 3 (adjusted odds ratio [AOR] = 10.54; 95% CI: 1.24–89.14, *p* = 0.031). Open defecation was also statistically associated with STH infections (AOR = 22.66; 95% CI: 1.18–433.58, *p* = 0.038). This study highlights a low prevalence of STH infections in the study area, with hookworm being the most common. The potential risk factors identified were linked to educational level and open defecation practices. Improving sanitation and enhancing health education to raise public awareness about hygiene are essential strategies for controlling STH infections among primary schoolchildren in remote areas.

## 1. Introduction

Soil-transmitted helminths (STHs) are identified as a group of neglected tropical diseases [1]. The most commonly identified parasites in human infections are *Ascaris lumbricoides*, *Trichuris trichiura*, and hookworms (*Necator americanus* and *Ancylostoma duodenale*) [2–4]. The World Health Organization (WHO) estimates that around 1.5 billion people worldwide are infected with STHs and highlights that school-age children are especially vulnerable to these infections [5]. STH infections in children have the potential to affect their physical, intellectual, and cognitive progress [2, 6, 7]. STH transmission and infection are influenced by a variety of variables, including age, geographical location, socioeconomic status, occupation, inadequate sanitation, and poor personal hygiene [2, 3].

Several studies in Thailand found that the frequency of STH infection among schoolchildren ranged from 5.6% to 75.1% [8–13]. The predominant types of STH infections differ among geographical regions. Hookworm infection has long been the most common parasitic illness in southern Thailand among people of all ages [14, 15]. In the 1980s, the prevalence of hookworm infection in southern Thailand reached as high as 76%. During this period, a national program introduced treatment with a single dose of mebendazole [16]. Twenty years later, in 2002, the prevalence of hookworm infection in children fell to 21.1%, owing to improved awareness of personal health and hygiene, as well as increased access to health care [17]. Our previous study conducted in Nakhon Si Thammarat revealed a 16% prevalence of intestinal parasites, with hookworms as the most common STH infection [18]. The Ministry of Public Health of Thailand recently reported that the overall prevalence of helminthiasis in the country is 9.79%. Hookworms are the most prevalent at 4.47%, followed by *Opisthorchis viverrini* at 2.2%, with hookworm infections particularly common in the southernmost region [19].

Children aged 7–12 years are at particular risk due to their characteristic behavioral patterns, such as frequent outdoor play and less developed hygiene practices, which contribute significantly to their heightened susceptibility. STH infections in this age group can lead to a range of negative health outcomes, including anemia, malnutrition, and impaired physical development, which may result in long-term cognitive deficits and reduced educational achievement [5]. The previous study also reported a high prevalence of STH infections among children in this age group in Southern Thailand [18]. However, data on STH infections in remote districts remain limited. Therefore, this study aims to evaluate the prevalence of STH infections and identify associated risk factors among schoolchildren in Koh Yao District, Phang Nga Province, Thailand.

## 2. Materials and Methods

### 2.1. Study Design and Setting

A school-based cross-sectional research was carried out at 12 schools in Koh Yao, Phang Nga Province, Southern Thailand, between January and August 2023. Koh Yao District is about 827 km south of Thailand's capital, Bangkok. Koh Yao District comprises three subdistricts, including Koh Yao Noi, Ko Yao Yai, and Phru Nai. The population of the district is approximately 14,655 in 2023 (Thailand Department of Provincial Administration). The district includes several islands in the Ko Yao archipelago in Phang Nga Bay, near Phuket. The two main islands are Ko Yao Yai (meaning “big long island”) and Ko Yao Noi (meaning “small long island”). Transportation to and from the district is facilitated by ferries and speedboats connecting Koh Yao to Phuket and Phang Nga provinces.

### 2.2. Ethical Considerations

Walailak University's Human Ethics Committee authorized the study prior to participant recruitment (Approval Number WUEC-22-335-01). Following a full description of the study's aims, participants' parents or legal guardians provided written informed permission.

### 2.3. Study Population and Sample Size

The research targeted children aged 7 to 12 years who attended primary schools in Koh Yao District. The sample size was obtained using the single proportion formula for an infinite population:

(1) $$\begin{array}{c} \frac{Z^{2}p\left(1-p\right)}{d^{2}}. \end{array}$$

If *p* is the prior study's prevalence of intestinal parasites, *d* is the margin of error and Z is the z-score that corresponds to the specified confidence level (CI) (1.96 for 95% CI). Previous studies revealed a prevalence rate of 19.8% [17] and a margin of error of 0.05, and a sample size of 244 was determined. Children who received intestinal helminthiasis therapy within 1 month of sample collection, opted out, or were unable to give stool samples on the scheduled day were excluded from the study. The samples were selected randomly from 12 primary schools.

### 2.4. Questionnaire Survey

A structured questionnaire was developed to collect demographic data (such as age, gender, and level of education), as well as information on potential risk factors. Local extension workers supervised the self-completion of these questionnaires to ensure data quality. Consistency and completeness were reviewed both on-site and again before data entry. In addition, two professional interviewers administered the questionnaire to the participating kids in person.

### 2.5. Stool Sample Collection

Each child was given verbal instructions on collecting and handling fecal samples. A clean plastic container was provided to each student a day prior to sample collection. Students collected a single fresh feces sample of 2–10 g in a clean, dry, leak-proof cup labeled with an identity number. The research team guided the students on providing a thumb-sized sample and explained the transport procedure. The students then submitted their labeled containers, which were checked to ensure correct labeling, sample quantity, and adherence to collection guidelines. The specimens were stored at a low temperature and promptly transported for examination.

### 2.6. Laboratory Procedure

Each stool sample was analyzed using the formalin-ethyl acetate sedimentation concentration method to detect parasites, even in low quantities [20]. About 1 g of each sample was treated with 10% formalin, filtered, centrifuged, and then mixed with ethyl acetate for further centrifugation. The sediment was examined under a microscope for parasite eggs or larvae using standard CDC methods [18]. Additionally, infection intensity was assessed using the modified Kato-Katz procedure [21]. Briefly, each stool specimen was homogenized by pressing it through a stainless steel sieve (40 mesh, 420 μm aperture) to remove large particulates. An aliquot of the sieved material was then transferred into a standardized template, yielding a fixed sample mass of approximately 39.2 mg. The sample was subsequently covered with a piece of cellophane presoaked in a glycerin–malachite green solution and compressed between the template and a glass slide to achieve uniform thickness. Following a clearing period of 30 min, the prepared slides were examined under a light microscope for the detection and quantification of parasitic eggs. Based on WHO criteria, hookworm infections were classified as low (1–1999 EPG), moderate (2000–3999 EPG), or high (≥ 4000 EPG) [22]. To avoid bias, two senior technologists, blinded to participant information, independently examined each sample.

### 2.7. Data Analysis

Data analysis was performed using SPSS Version 20. Descriptive statistics summarized the characteristics of participants. Logistic regression was applied to examine the relationship between independent variables and STH infections. Univariate analysis was conducted to explore crude associations between the binary outcome (hookworm infection vs. no hookworm infection) and each independent variable. All variables identified in the univariate analysis were considered relevant and subsequently included in a multiple logistic regression using the entire method to adjust for potential confounding factors. The final model results were reported with adjusted odds ratios (AOR) and 95% CIs, with statistical significance set at a *p* value of less than 0.05.

## 3. Results

### 3.1. Demographic Characteristics

A total of 243 schoolchildren were included in the study. Approximately half of the participants were males, accounting for 125 (51.44%) of the total study population. The age of study participants ranged from 7 to 12 years, with a mean of 8.99 ± 1.57 years. A total of 131 schoolchildren (53.91%) were in Grades 1–3, while 112 schoolchildren (46.09%) were in Grades 4–6. The predominant religious group was Muslim, comprising 91.3% of the participants (Table 1).

### 3.2. Prevalence of STH Infections

Among the 243 schoolchildren surveyed, the overall prevalence of intestinal parasitic infections was 4.94% (12/243; 95% CI: 2.60%–8.50%), with boys exhibiting higher infection rates than girls. Hookworm accounted for the majority of the infections (4.11%; 10/243), followed by *T. trichiura* (1.65%; 4/243). No other parasitic species were found in the study samples (Table 2). Ten of the 243 schoolchildren had single infections (eight hookworm infections and two *T. trichiura* infections), and two children were found to have coinfections of *T. trichiura* and hookworm. The mean eggs per gram (EPG) were 632.5 for hookworm and 460 for *T. trichiura*. While most hookworm infections were classified as light, one case was categorized as moderate. In terms of geographical setting, positive cases were found to be higher in Koh Yao Yai district compared to other areas (Table 2).

### 3.3. Health Behaviors Related to Intestinal Parasitic Infections

The study results revealed varying health behaviors among participants. Handwashing after defecation was the most consistently practiced behavior, with 89.71% always doing so. About 79.01% of participants never ate undercooked food, whereas only 2.47% always ate undercooked or raw food. Handwashing before meals was relatively common at 37.86%, although 60.91% did so only sometimes. A majority (83.95%) always wore shoes outside, whereas 51.85% always played on dirt or grass. Of interest, only 16.05% always ate raw vegetables, whereas 67.08% ate them sometimes. Overall, most participants exhibited generally good hygiene habits, especially in handwashing after defecation and wearing shoes outside. However, some risky behaviors were still observed. For example, 51.85% of participants reported sometimes touching pets, and 43.62% reported sometimes playing on dirt or grass (Table 3).

### 3.4. Independent Variables and Their Associations With STH Infections

Our study analyzed the association between various characteristics and the presence of STH infections among participants. Female participants had a slightly lower infection rate (3.39%) compared to males (6.40%), but the crude odds ratio (COR) indicated no significant difference (COR = 0.51, 95% CI: 0.15–1.75, *p* = 0.42). Age did not significantly affect infection rates, with similar prevalence observed in both age groups. Education level was a significant factor, where children in grades higher than three showed notably increased odds of infection (AOR = 10.54, 95% CI: 1.24–89.14, *p* = 0.031). Contact with domestic animals and handwashing before meal did not show a significant association with STH infections. Handwashing after defecation (AOR = 0.39, 95% CI: 0.16–2.62, *p* = 0.332) and after animal contact (AOR = 0.32, 95% CI: 0.07–1.52, *p* = 0.152) showed a tendency toward being protective factors against STH infections; however, these associations were not statistically significant. Interestingly, open defecation was strongly associated with higher infection rates, marked by a significantly increased odds ratio (AOR = 22.66, 95% CI: 1.18–433.58, *p* = 0.038) compared to those using toilets. These results underscore the importance of educational interventions and sanitation improvements, particularly focusing on young schoolchildren and practices such as open defecation (Table 4).

## 4. Discussion

Findings from this study indicate that among the 243 schoolchildren surveyed, 12 (4.94%) were infected with at least one type of STHs. The prevalence recorded in this study is greater than that discovered in similar research undertaken in Thailand over the previous two decades, such as the 4.24% prevalence reported in central Thailand, which includes Ang Thong, Ayutthaya, and Suphan Buri Provinces in 2004 [23]. However, the overall rate of STH infections in this research is lower than those in earlier investigations conducted in southern Thailand. This study's prevalence is less than the 75.1% recorded in Narathiwat Province in 2003 [9], the 19.8% discovered in a nationwide survey of southern Thailand in 2009 [15], and the 16% reported in rural parts of Nakhon Si Thammarat in 2016 [18]. Our findings are consistent with the current status of helminth infections in the country conducted by the Ministry of Public Health of Thailand, which reported an overall prevalence of 9.79% in 2019 [19]. This reduction in STH infections, especially hookworms, may be attributed to various factors, such as improvements in sanitation and enhanced health education initiatives aimed at promoting better hygiene practices. Despite these positive trends, our study revealed a prevalence of 4.94% among primary school children in Koh Yao District, indicating that STH infections continue to be a significant public health concern in southern Thailand. The persistence of these infections underscores the importance of continuous monitoring and focused interventions, especially in rural areas where healthcare resources may be scarce. These findings suggest that, despite a decline over the past two decades, intestinal parasitic infections remain a notable public health concern requiring continued attention.

Hookworm was the most frequently detected helminth among schoolchildren, consistent with findings from previous studies conducted in southern Thailand [15–17]. The hookworm infection at 4.11% in our study is significantly lower than rates reported in earlier studies from southern Thailand, where the infection rate was 21.1% in 2002 [17]. Additionally, national surveys showed a prevalence of 15.8% in 2009 [15] and 4.47% in 2019 [19]. Of interest, the low prevalence of *T. trichiura* (1.65%) in this study compares with study findings from rural southwestern Kenya (0.6%) [24], Harbu Town, Northeastern Ethiopia (0.4%) [25], and Northwest Ethiopia [26]. The low prevalence observed in our study likely aligns with participants' health behaviors, particularly good hygiene practices such as handwashing after defecation and wearing shoes outdoors. Furthermore, the low prevalence may reflect the influence of environmental and infrastructural changes in Thailand. Improvements in sanitation infrastructure, such as increased access to clean water, household latrines, and waste management systems, have likely played a critical role in interrupting the transmission cycle of STHs. Despite the declining prevalence, our findings revealed that hookworm remained the most common parasitic illness among schoolchildren in the study area.

To assess the risk factors for STH infection, we conducted multivariate analysis, which revealed a statistically significant association between education level and open defecation. Specifically, children in grades higher than three exhibited a greater prevalence of intestinal parasites compared to those in Grades 1 to 3. These findings are congruent with research carried out in rural areas in Nakhon Si Thammarat, Thailand [18], and are also in agreement with other research findings [27–30]. The observed association between higher grade levels and STH infections may reflect a complex interplay of behavioral and environmental factors. Older children may engage in more independent activities in potentially contaminated areas. In addition, peer influences, or reduced parental supervision may also contribute to a higher rate of infections in this age group. Additionally, open defecation was significantly associated with STH infections. These findings are in line with research from Ethiopia, where a high prevalence of intestinal parasites was similarly linked to factors such as inadequate awareness of parasitic infections, open defecation, and the consumption of unwashed raw vegetables [31–33]. However, the extremely wide CI for open defecation (1.18–433.58) is directly attributed to the small number of individuals (*n* = 5) who engaged in open defecation. Therefore, the statistical model has limited data to precisely estimate the relationship between STH infections and open defecation. To obtain more precise estimates of the association between open defecation and STH infections, further studies with larger sample sizes are crucial, particularly those ensuring adequate representation of individuals practicing open defecation. Given these findings, we recommend implementing targeted health education programs that focus on improving hygiene practices and promoting safe sanitation methods among schoolchildren. Encouraging proper handwashing techniques and reducing open defecation through community awareness campaigns can significantly help decrease the prevalence of STH infections. Future research should explore longitudinal studies to monitor changes in parasitic infection rates over time and assess the effectiveness of educational interventions on reducing infection rates. Additionally, investigating the socioeconomic factors that contribute to STH prevalence in various subdistricts could provide deeper insights and lead to more tailored public health strategies.

This study had several limitations, including the reliance on a single-day fecal examination to detect helminths, which may impact diagnostic sensitivity. The seasonality of parasitic infections was not assessed, so the disease prevalence reported in this study does not account for this factor. Additionally, the risk factor analysis was based solely on questionnaire data, potentially omitting other relevant factors. This study employed a cross-sectional design and was conducted in a single rural district, which may not account for seasonal or temporal variations in infection rates. Consequently, the findings may have limited generalizability to schoolchildren in urban areas or other regions with differing environmental conditions and sanitation practices. Moreover, socioeconomic status was not included as a variable in this study, despite its potential contribution to the prevalence of STH infections. The study's strengths include the use of the formalin-ethyl acetate concentration technique and the modified Kato-Katz method for parasite detection, both conducted by experienced medical laboratory technologists, ensuring accurate results. The relatively large sample size further strengthens the study, along with the successful collection of specimens and questionnaires from young children in rural communities, which provided valuable insights into the issue of intestinal parasitic infections. Future research should employ longitudinal study designs to clarify causal relationships and capture temporal trends in STH transmission. In addition, the inclusion of detailed socioeconomic variables—such as household income and parental education—may help identify underlying factors associated with infection risk.

## 5. Conclusions

This study highlights the prevalence of STH infections among primary schoolchildren, revealing an overall 4.94% infection rate, predominantly due to hookworm in Koh Yao District, Phang Nga Province. Despite generally good hygiene practices observed among participants, certain behaviors, such as touching pets and playing in the dirt, may contribute to the persistence of parasitic infections. Our findings suggest that while progress has been made in reducing the prevalence of intestinal parasites, public health interventions are still necessary.

## Figures and Tables

**Table 1:** Demographic profile of participants (*n* = 243).

Characteristics	Number	Percentage
Gender		
Male	125	51.44
Female	118	48.56
Level of education (grade)		
1	59	24.28
2	42	17.28
3	30	12.35
4	31	12.76
5	63	25.93
6	18	7.41
Religion		
Buddhist	21	8.64
Muslim	222	91.36

**Table 2:** Prevalence of infection among schoolchildren in Koh Yao, Phang Nga Province, Thailand (*n* = 243).

	Number of positive	%
Parasite species		
*Trichuris trichiura*	4	1.65
Hookworm	10	4.11
Type of infection		
Single infection	10	4.11
Coinfection	2	0.82
Setting		
Koh Yao Noi	2	0.82
Koh Yao Yai	6	2.47
Phru Nai	4	1.64
Total	12	4.93 (2.60, 8.50)

**Table 3:** Health behaviors related to parasitic infections among primary schoolchildren in Koh Yao, Phang Nga Province, Thailand (*n* = 243).

Behavior	Practice (number, %)		
	Always	Sometimes	Never
Handwashing before meals	92 (37.86)	148 (60.91)	3 (1.23)
Handwashing after defecation	218 (89.71)	23 (9.47)	2 (0.82)
Handwashing after pet contact	95 (39.09)	125 (51.44)	23 (9.47)
Handwashing after playing outside	97 (39.92)	127 (52.26)	19 (7.82)
Eating undercooked/raw food	6 (2.47)	45 (18.52)	192 (79.01)
Eating raw vegetables	39 (16.05)	163 (67.08)	41 (16.87)
Using toilet at home	229 (94.24)	9 (3.70)	5 (2.06)
Touching pets	73 (30.04)	126 (51.85)	44 (18.11)
Wearing shoes outside	204 (83.95)	38 (15.64)	1 (0.41)
Playing on dirt or grass	126 (51.85)	106 (43.62)	11 (4.53)

**Table 4:** Factors associated with STH infections among primary schoolchildren in Koh Yao, Phang Nga Province, Thailand (*n* = 243).

Characteristics	*n*	STH	COR (95% CI)	*p* value	AOR (95% CI)	*p* value
Gender						
Male	125 (51.44)	8 (6.40)	Ref.	0.287	Ref.	0.217
Female	118 (48.56)	4 (3.39)	0.51 (0.15–1.75)		0.42 (0.10–1.67)	
Age group						
≤ 9	141 (58.02)	7 (4.96)	Ref.	0.982	Ref.	0.116
> 9	102 (41.98)	5 (4.90)	0.99 (0.30–3.20)		0.181 (0.02–1.53)	
Education level						
≤ Grade 3	131 (53.91)	5 (23.82)	Ref.	0.387	Ref.	0.031^∗^
> Grade 3	112 (46.09)	7 (6.25)	1.68 (0.52–5.45)		10.54 (1.24–89.14)	
Contact with domestic animals						
No	103 (42.39)	6 (5.83)	Ref.	0.586	Ref.	0.407
Yes	140 (57.61)	6 (4.29)	0.72 (0.23–2.31)		0.56 (0.14–2.21)	
Handwashing before meals						
No	151 (62.14)	6 (3.97)	Ref.	0.379	Ref.	0.639
Yes	92 (37.86)	6 (6.52)	1.69 (0.53–5.40)		1.41 (0.33–6.00)	
Handwashing after defecation						
No	25 (10.29)	2 (8.00)	Ref.	0.462	Ref.	0.332
Yes	218 (89.71)	10 (4.59)	0.55 (0.11–2.68)		0.39 (0.16–2.62)	
Handwashing after animal contact						
No	148 (60.91)	8 (5.41)	Ref.	0.676	Ref.	0.152
Yes	95 (39.09)	4 (4.21)	0.77 (0.23–2.63)		0.32 (0.07–1.52)	
Eating raw or undercooked food						
No	192 (79.01)	10 (5.21)	Ref.	0.707	Ref.	0.492
Yes	51 (20.99)	2 (3.92)	0.74 (0.16–3.50)		0.55 (0.10–3.00)	
Eating fresh vegetables						
No	41 (16.87)	2 (4.88)	Ref.		Ref.	0.496
Yes	202 (83.13)	10 (4.95)	1.02 (0.21–4.82)		1.89 (0.30–11.83)	
Open defecation						
Yes	5 (2.06)	1 (20.0)	5.519 (0.53–50.10)	0.157	22.66 (1.18–433.58)	0.038^∗^
No	238 (97.94)	11 (4.62)	Ref.		Ref.	

*Note:* CI: 95% confidence interval.

Abbreviations: AOR, adjusted odd ratio; COR, crude odd ratio.

^∗^Significant association.

## Data Availability

All the data related to this study are available from the corresponding author upon request.
